# A Comparison of Three Child OHRQoL Measures

**DOI:** 10.3390/dj7010019

**Published:** 2019-02-12

**Authors:** Lyndie Foster Page, Fiona Gilchrist, Hillary L. Broder, Ellen Clark, W. Murray Thomson

**Affiliations:** 1 Faculty of Dentistry, Otago University, Dunedin 9016, New Zealand; lyndie.fosterpage@otago.ac.nz; 2 School of Clinical Dentistry, The University of Sheffield, Sheffield S10 2TA, UK; f.gilchrist@sheffield.ac.uk; 3 Cariology and Comprehensive Care, NYU College of Dentistry, 137 East 25th Street, 5th floor, New York, NY 10010, USA; hillary.broder@nyu.edu; 4 Oral Health Service Northland District Health Board Ward 5, Whangarei Hospital Private Bag 9742, Whangarei 0148, New Zealand; Ellen.Clark@northlanddhb.org.nz

**Keywords:** quality of life, children, oral health, measurement

## Abstract

Comparing oral health-related quality of life (OHRQoL) measures can facilitate selecting the most appropriate one for a particular research question/setting. Three child OHRQoL measures Child Perceptions Questionnaire (CPQ_11–14_), the Child Oral Health Impact Profile (COHIP) and the Caries Impacts and Experiences Questionnaire for Children (CARIES-QC) were used with 335 10- to 13-year-old participants in a supervised tooth-brushing programme in New Zealand. The use of global questions enabled their validity to be examined. Assessments were conducted at baseline and after 12 months. All three measures had acceptable internal consistency reliability. There were moderate, positive correlations among their scores, and all showed differences in the impact of dental caries on OHRQoL, with children with the highest caries experience having the highest scale scores. Effect sizes were used to assess meaningful change. The CPQ_11–14_ and the CARIES-QC showed meaningful change. The COHIP-SF score showed no meaningful change. Among children reporting improved OHRQoL, baseline and follow-up scores differed significantly for the CPQ_11–14_ and CARIES-QC measures, although not for the COHIP-SF. The three scales were broadly similar in their conceptual basis, reliability and validity, but responsiveness of the COHIP-SF was questionable, and the need to compute two different scores for the CARIES-QC meant that its administrative burden was considerably greater than for the other two measures. Replication and use of alternative approaches to measuring meaningful change are suggested.

## 1. Introduction

Oral health was defined by Locker as “a standard of the oral tissues which contributes to overall physical, psychological and social well-being by enabling individuals to eat, communicate and socialise without discomfort, embarrassment or distress and which enables them to fully participate in their chosen social roles” [1]. To date, a great deal of research effort has focused on developing, validating and testing what are usually referred to as “OHRQoL measures” but are scales which measure the impact of oral conditions on people’s lives. After the initial work focused on measures for adults, attention turned to the more difficult task of developing and validating scales for use with children (and their parents) [2], and these have now been in use for well over 15 years. Most scales measure only negative aspects (impacts) of oral health, thereby failing to encompass the positive aspects which are more congruent with current definitions of oral health-related quality of life (OHRQoL) [3]. The two most commonly used child OHRQoL measures are the short-form versions of the CPQ_11–14_ [4] and the COHIP [5]. These were developed for use with a wide range of conditions which affect children’s day-to-day lives, such as dental caries, tooth loss, malocclusion and orofacial developmental defects. A more recently developed measure is the CARIES-QC [6], developed with the intention of producing a condition-specific and responsive instrument for use in clinical studies of children with dental caries.

Where competing measures exist (such as the CPQ_11–14_, the COHIP and the CARIES-QC), it is important to compare their properties and identify important differences, so that the most appropriate measure can be selected for a particular research question or setting [7,8,9]. Undertaking such a comparison should ideally use a systematic approach, using a set of established criteria, such as the eight important attributes of self-report measures described by the Scientific Advisory Committee of the Medical Outcomes Trust [9]. Those are having a conceptual model, reliability, validity, responsiveness, interpretability, respondent and administrative burden, alternative forms, and cross-cultural applicability. Often, such measures take years to be validated and replicated in diverse populations. Being underpinned by a conceptual model means that a measure is based on a thorough understanding of the entity which is being measured [10]. Assessing reliability involves considering both repeatability (the stability of measurements over time—assuming the entity being measured has not changed during that time—and intra- and inter-rater agreement) and precision, which encompasses the intercorrelation of the various items comprising a multi-item scale. Validity is essentially the degree to which the instrument measures what it purports to measure (including relevant domains, appropriate score gradients across ordinal categories of a “gold standard” measure, and being able to relate the score range to theoretical understanding of the construct being measured, along with appropriate interpretation). Responsiveness is the scale’s ability to reflect meaningful change in that construct, whereas the criterion of interpretability requires that the scores themselves should have meaning. The notions of respondent and administrative burden require that it should be neither too long nor too difficult to use, whereas having alternative forms is closely related to the former, in that having a short-form version both minimises respondent burden and makes it more likely that the instrument will be used. Finally, the property of cross-cultural applicability is important for enabling comparisons of different populations.

That set of criteria makes a useful framework for evaluating and comparing child OHRQoL measures. The aim of this study was to examine and compare the properties of the 16-item CPQ_11–14_, the COHIP-SF and the CARIES-QC in a longitudinal study of New Zealand children. 

## 2. Method

A survey was conducted of 335 10- to 13-year-old children attending for dental treatment in Northland community clinics in 2015 as part of a supervised tooth brushing programme [11]. Ethical approval for the study was given from the Northern A Health and Disability Ethics Committee (14/NTA/176). Consent was obtained from both parent and child before proceeding. 

### 2.1. Sociodemographic Characteristics

Information was gathered on each child’s sex, age and ethnicity. An area-based deprivation measure [University of Otago, 2013] was used to allocate each participant to a deprivation decile score, based on the residential address of the child’s household. Areas with scores 1 to 3 were classified as “low deprivation”; those with scores 8 to 10 were classified as “high deprivation”.

### 2.2. OHRQoL Measures

Oral health-related quality of life was measured using the recently modified 16-item CPQ_11–14_ questionnaire [12], the 19-item COHIP-SF [13] and the newly developed 12-item CARIES-QC [5]. The study questionnaire was designed so that the three OHRQoL scales (presented in the following order: CPQ_11–14_, COHIP-SF and CARIES-QC) were separated by a number of questions on the child’s oral hygiene practices. The item content of the three OHRQoL measures is summarised in Table 1. The reference period used for the CPQ and COHIP-SF is the previous three months, whereas for the CARIES-QC, the items referred to the time of examination. The CPQ_11–14_ includes 16 items grouped into two domains represented by the ‘symptoms/function’ and ‘well-being’ (combined emotional and social well-being) subscales [10]. Its item response options and scores are: ‘Never’ (scoring 0); ‘Once or twice’ (1); ‘Sometimes’ (2); ‘Often’ (3); and ‘Every day or almost every day’ (4). The COHIP-SF includes 19 items which represent the three domains of oral health (five items), functional well-being (four items) and socio-emotional well-being (ten items). For each question, participants are asked how frequently they have experienced an experience/impact relating to their teeth, mouth or face. Response options and scores are: ‘Never’ (scoring 0); ‘Almost never’ (1); ‘Sometimes’ (2); ‘Fairly often’ (3), and ‘Almost all of the time’ (4). The COHIP-SF contains items to assess both positive and negative aspects of OHRQoL. In the current study, the positive items were reversed at the time of analysis, in order for a higher score to reflect poorer OHRQoL (as with the CPQ_11-14_). This differs from the original measure, where a higher score reflects better OHRQoL. The CARIES-QC contains 12 items in one domain relating to how caries specifically impacts on a child’s OHRQoL. Response options and scores are: ‘Not at all’ (scoring 0); ‘A bit’ (1), and ‘A lot’ (2).

The standard global self-reported oral health questions for each scale were also used, so that their validity could be examined. For the CPQ_11–14_, children were first asked to rate the health of their teeth, lips, jaws and mouth (response options: “Excellent’, ‘Very good’, ‘Good’, ‘OK’ or ‘Poor’). Second, they were asked how much their teeth, lips, jaw or mouth affects their life overall (response options: ‘Not at all’, ‘A little bit’, ‘Some’, ‘A lot’, ‘Very much’). For the COHIP-SF, they were asked to rate the health of their teeth, lips, jaws and mouth (response options: ‘Excellent, ‘Good’, ‘Average’, ‘Fair’ or ‘Poor’); for the CARIES-QC, they were asked “How much of a problem are your teeth for you?” (response options: ‘Not at all’, ‘A bit’ or ‘A lot’).

### 2.3. Clinical Measures

The International Caries Detection Assessment (ICDAS) index was used to record a restoration score and then a caries score for each surface of every tooth [14]. An experienced dentist undertook all of the clinical examinations, having been trained in the study protocol at one of the five community clinics. The examiner was calibrated in the use of ICDAS prior to examining all the children. A standardised approach was used for all clinical examinations. The child was reclined partially on the clinic chair and the examiner was seated behind the child. A standard LED headlight was used for all clinical examinations. The teeth were first charted as unerupted, missing or present, as well as whether they were primary or permanent. The examination commenced beginning with the most distal molar in the 1st quadrant, moving around to the last molar in the 2nd quadrant, and following on with the 3rd and 4th quadrants. Teeth were first examined wet, before air drying, as required by the ICDAS protocol. The data were recorded manually on a standard ICDAS scoring sheet. As well as the ICDAS scoring, a conventional DMFS/dmfs score was computed for each child, using an ICDAS code 3 or higher [15]. 

Posterior bitewing radiographs were taken before the clinical dental examination. These were read later and a separate radiographic diagnosis data-set compiled. This was later merged with the clinical caries status data-set and used to adjust (where appropriate) the caries status of the posterior teeth. Traditional DMFT and dmft scores were then calculated and, because all children were in the mixed dentition phase, the DMFT and dmft for each child were then combined to give an overall level of caries experience score. Repeat clinical examinations were conducted on 33 children by one examiner. The intraclass correlation coefficient was 0.85, indicating acceptable intra-examiner reliability.

### 2.4. Follow-up Data Collection

Approximately one year later, a repeat data collection took place, capturing similar data to those collected at baseline. 

### 2.5. Data Analysis

Data were analysed with SPSS (version 23.0). The analysis commenced with the computation of scale scores, after which summary statistics for dental caries experience were produced. The scales’ validity was determined using baseline scores. Internal consistency reliability was assessed using Cronbach’s alpha. Pearson’s r was used to examine the correlations among scores on the CPQ_11–14_, COHIP-SF and CARIES-QC scales. Test-retest reliability of scale scores was assessed for a re-examined subset of participants by using Intraclass Correlation Coefficients (ICC). Cross-sectional construct validity was evaluated by examining the association between the rating of how much the child’s oral condition affected his/her overall well-being and the mean scale scores. Mann–Whitney or Kruskal–Wallis tests were used (as appropriate) for comparing scores for continuous variables (where these were not normally distributed). 

The CPQ_11–14_, COHIP-SF and CARIES-QC scores at baseline and follow-up were calculated, along with the change in those scores (by subtracting follow-up scores from baseline scores, so that a positive change score indicates an improvement in OHRQoL, and a negative one represents deterioration). Because longitudinal use of the CARIES-QC scale requires the conversion of the raw scale score to an interval score (and then the use of both in the description of the change in score after an intervention), we used both in the analysis of the follow-up data in this study. Paired t-tests were used to test the statistical significance of scale score changes, and the clinical significance or meaningful magnitude of change was determined by the calculation of effect sizes. Effect-size statistics were calculated by dividing the mean change score by the standard deviation of the baseline score, in order to give a dimensionless measure of effect for each scale (where effect-size statistics of <0.2 indicate a small clinically meaningful magnitude of change, 0.2–0.7 a moderate change, and >0.7 a large change). 

Longitudinal construct validity was evaluated in a number of ways, but first by scrutinising the association between change scores. As used previously [16], acceptable longitudinal construct validity is apparent where individuals reporting deterioration have negative mean change scores, those reporting stability have change scores of approximately zero, and those reporting improvement have positive change scores. Paired t-tests were used to examine the significance of the within-individual change of those who changed and those for whom stability was reported. If the former is statistically significant and the latter not, there is support for the assertion that the measure is responsive. Finally, the mean change scores of those for whom ‘a little’ improvement was reported were used to determine the minimally important difference for each of the CPQ_11–14_, COHIP-SF and CARIES-QC scales. 

## 3. Results

The 335 10- to 12-year-olds (51.6% female) who took part in the study represent a 78.4% participation rate. Two-thirds were Māori, and more than three-quarters (76.4%) resided in highly deprived areas. The overall mean DMFT/dmft was 2.1 (SD, 2.4 range 0–13). More than two-thirds of the children presented with carious teeth, and one-fifth had more than 4 teeth affected.

### 3.1. Cross-sectional Reliability and Validity

**Figure 1:**
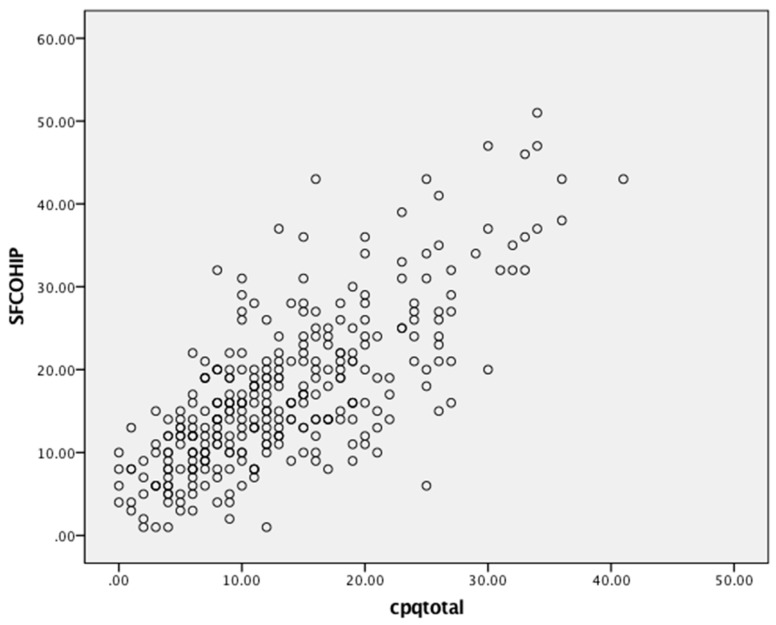
Scatterplot of baseline COHIP-SF and CPQ_11-14_ scale scores.

**Figure 2:**
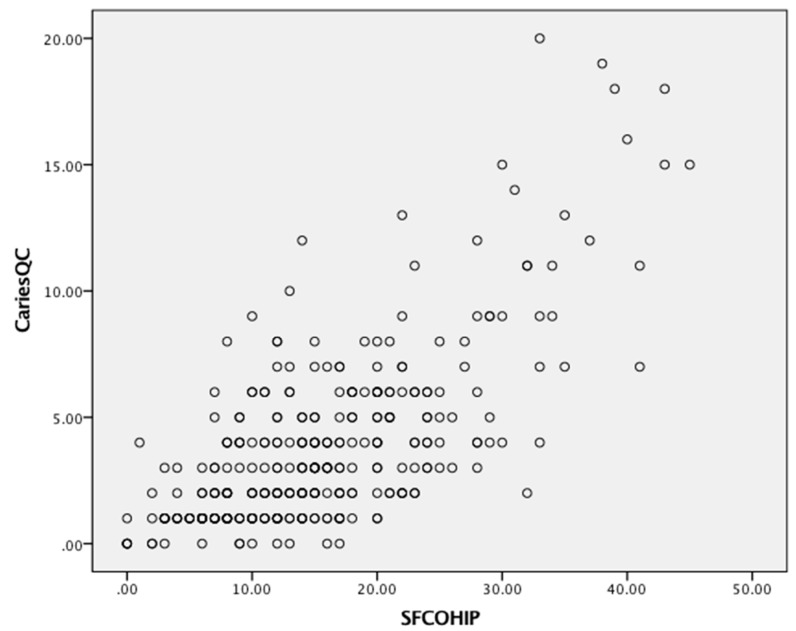
Scatterplot of baseline COHIP-SF and CARIES-QC scale scores.

**Figure 3:**
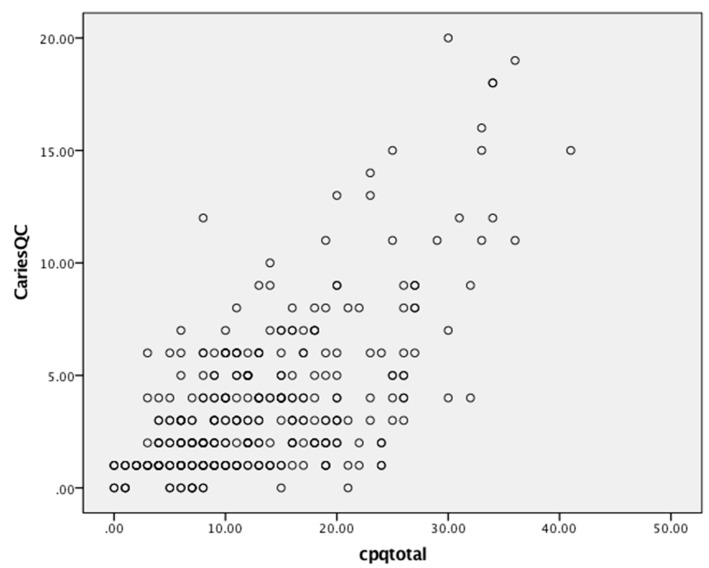
Scatterplot of baseline CPQ_11-14_ and CARIES-QC scale scores.

Scores ranged from 0 to 40, 1 to 51 and 0 to 24 for the CPQ_11–14_, COHIP-SF and CARIES-QC, respectively (Table 2). All three measures detected substantial variability in children’s OHRQoL, as shown by their scores. Floor effects ranged from 0 to 24%, and ceiling effects were observed only with the CARIES-QC. Substantial internal consistency reliability was apparent for each of the three questionnaires (with Cronbach’s alpha values all in the acceptable range of 0.70–0.90, but that of the COHIP-SF and CARIES-QC being slightly superior to that of the CPQ_11–14_). There was a moderate and positive correlation between scores on the CPQ_11–14_ and the COHIP-SF (Figure 1), and the CARIES-QC and COHIP-SF (Figure 2) with a Pearson’s r of 0.71 for both. A similar moderate but slightly lower positive correlation was found with the CPQ_11–14_ and CARIES-QC (Figure 3) with a Pearson’s r of 0.64. Assessment of test-retest reliability was undertaken with 38 participants at baseline, with ICCs of 0.82, 0.78 and 0.80, respectively, for the CPQ_11–14,_ COHIP-SF and the CARIES-QC.

All three measures showed differences in the impact of dental caries on quality of life (although the differences in scores was not statistically significant), with the greatest scores in the expected direction: children who presented with the highest caries burden had the highest scores (Table 3). 

All measures showed statistically significant higher scores among those with poorer self-rated oral health, whether it was oral health per se and/or overall impact on quality of life (Table 4). We observed the expected gradients in mean scale scores (with higher scale scores among those reporting poorer oral health) across the global item response categories.

### 3.2. Follow-up and Responsiveness

Of the 335 children who were examined at baseline, there were 95 (28.4%) who did not have follow-up examinations. Table 5 compares baseline sociodemographic characteristics and scale scores of children followed up and lost to follow-up. There were significantly more Māori children who were not followed up. The group who were not followed up had higher scores for the CPQ_11–14_ and the CARIES-QC.

Data on the baseline and follow-up scores are presented (with effect-size statistics) in Table 6. Effect sizes showing moderate to meaningful changes were associated with statistically significant reductions in scores in the CPQ_11–14_ and the CARIES-QC. The COHIP-SF score showed no meaningful change from baseline to follow-up.

Among those who improved, baseline and follow-up scores differed significantly (determined by paired t-tests) for the CPQ_11-14_ and CARIES-QC measures, although not for the COHIP-SF (Table 7). In all cases, the follow-up score was lower than the baseline score. Among those who remained stable, the differences between the baseline and follow-up scores for the CPQ_11–14_ and CARIES-QC scales were significantly different, but not for the COHIP-SF, which failed to reflect the improvement in OHRQoL which was evident with the other two scales. For those who deteriorated, baseline and follow-up scores differed but not significantly for any of the measures, although in all cases the follow-up score was higher than the baseline score.

The minimal important difference (equivalent to the mean change score of those for whom a little improvement was reported) was 3.9 for the CPQ_11–14_, 2.0 for the COHIP-SF, and 1.4 (for the raw score) and 1.7 (for the interval score) for the CARIES-QC. Using the effect size methodology, we then computed the percentage of individuals showing or exceeding the minimal important difference by each of the measures. Overall, just over one-third of participants showed or exceeded the minimal important difference for the CPQ_11–14_ and the COHIP-SF (37.9% and 35.8%, respectively), whereas nearly one-third (32.9%) did so for the CARIES-QC (using the interval data). There were no significant differences by sociodemographic characteristics.

## 4. Discussion

This study set out to examine and compare the properties of three child OHRQoL measures in a longitudinal study of New Zealand children. It found that the CPQ_11–14_, the COHIP-SF and the CARIES-QC were broadly similar in terms of their conceptual basis, reliability and validity, but that there were two important differences: the responsiveness of the COHIP-SF was questionable, and the need to compute two different sets of scores for the CARIES-QC meant that its administrative burden was considerably greater than that of the other two scales. 

Consideration of the study’s weaknesses and strengths is appropriate before discussing the findings. Where the weaknesses are concerned, we did not measure all of the influences on a child’s OHRQoL, such as malocclusion; the focus of the study was on dental caries. From a study design perspective, this was an opportunistic study, using data from an interventional study which was set up primarily to answer other research questions [11]. Moreover, the CARIES-QC is not yet an established scale, given that it has only recently been published in the peer-reviewed scientific literature [6]. Another weakness is that, because we did not randomly mix up the order of the scales in the study questionnaire, it may be that responses to the COHIP-SF were unduly affected by respondent fatigue or short attention spans, and that may have affected the validity and reliability of the findings. Moreover, we did not use a global transition judgement to determine change and so had to construct a change indicator from responses to the global item at baseline and follow-up (each of which would have been susceptible to a degree of measurement error). Thus, our responsiveness data may not actually be accurate, and the comparison of the three scales may lack validity as a consequence [17,18]. Turning to the strengths of the study, the longitudinal design (enabling examination of responsiveness), the sample’s ethnic heterogeneity, and the concurrent use of three different OHRQoL scales are innovative and useful.

In comparing the three measures, considering them against the SAC criteria (Table 8) shows that there were similarities and differences. All three had acceptable reliability and validity, with floor and ceiling effects not apparent, and so any of those three scales would be appropriate for a cross-sectional investigation of dental caries experience and OHRQoL. However, the COHIP-SF failed to show acceptable responsiveness (particularly for those whose OHRQoL improved), so this should be investigated further in other longitudinal studies using a valid global transition judgement. The administrative burden was greater for the CARIES-QC because it required computation of an interval score. This complicates the analysis of the scale data. The rationale for using the interval score was that, as the scale focuses on attributes which are not directly measurable, the raw score represents a rank along the scale, and so the addition or subtraction of raw scores is not possible [19]. However, it could be argued that such “measurement theory fundamentalism” leads to an unnecessary and somewhat artificial analytical step, given that the correlation between the raw and interval scores in our sample was 0.96. It is likely that the effects of random error will have been greater than any systematic error arising from using the raw score instead of the interval score. Indeed, it could be argued that the issue has been superseded by common practice—there is a parallel in the debate about using item weights [20]—and the requirement for such instruments to be user-friendly and scores to be interpretable. 

In conclusion, this investigation has shown that the CPQ_11–14_, the COHIP-SF and the CARIES-QC would all be appropriate for use in cross-sectional investigations of dental caries experience and OHRQoL, but the longitudinal utility of the COHIP merits further investigation, using more appropriate study designs, change measures and replication. 

## Figures and Tables

**Table 1:** Item content of the CPQ_11–14_-ISF:16, SFCOHIP and CARIES-QC scales.

CPQ_11–14_ Items	COHIP-SF Items	CARIES-QC Items
*Symptoms domain*	*Oral health well-being domain*	
Pain in teeth/mouth	Pain in teeth	Teeth hurt
Bad breath	Bad breath	Hard to eat some foods
Mouth sores	Crooked teeth or spaces	Eat on one side of mouth
Food caught between teeth	Discoloured teeth	Food stuck
Difficulty saying words	Bleeding gums	Kept awake
Difficulty chewing firm foods	*Functional well-being domain*	Annoy you
Taken longer to eat a meal	Difficulty keeping teeth clean	Hurt when brush teeth
Difficulty eating/drinking hot/cold foods	Difficulty eating foods you like	Cried because of teeth
*Well-being domain*	Difficulty saying words	Hard to do school work
Upset	Trouble sleeping	Eat more carefully
Felt irritated/frustrated	*Socio-emotional well-being domain*	Eat more slowly
Felt shy	Avoided smiling/laughing	Cross because of teeth
Concerned what people think about teeth/mouth	Teased or bullied	
Teased/called names	Felt you look different	
Avoided smiling/laughing	Not wanting to speak/read aloud in class	
Asked questions	Worried or anxious	
Argued with children/family	Worried what people think about teeth/mouth	
	Missed school	
	Unhappy or sad	
	Felt attractive	
	Felt confident	

**Table 2:** Baseline descriptive statistics and internal consistency reliability data for the CPQ_11–14_, COHIP-SF and CARIES-QC and subscales.

Measures	Number of Items	Mean Score (SD)	Cronbach’s Alpha (α)	Range of Observed Scores	Percentage with Score 0	Percentage with Maximum Score
**CPQ_11–14_**	16	13.1 (8.0)	0.80	0 to 41	1.5	0.0
Subscales						
Symptoms	8	7.2 (4.1)	0.62	0 to 24	2.1	0.0
Well-being	8	6.0 (4.9)	0.75	0 to 21	12.8	0.0
**COHIP-SF**	19	17.1 (9.3)	0.83	1 to 51	0.0	0.0
Subscales						
Oral health well-being	5	5.3 (3.3)	0.62	0 to 17	5.1	0.0
Functional well-being	4	2.5 (2.4)	0.57	0 to 12	24.5	0.0
Socio-emotional well-being	10	9.2 (5.4)	0.74	0 to 30	1.5	0.0
**CARIES-QC**	12	3.8 (3.5)	0.83	0 to 24	5.1	0.3

**Table 3:** Mean CPQ_11–14_, COHIP-SF and CARIES-QC scores by sociodemographic characteristics and caries experience.

Characteristic	N (%)	Mean CPQ (SD)	Mean COHIP-SF (SD)	Mean CARIES-QC (SD)
Total	335 (100.0)	13.1 (8.0)	17.1 (9.3)	3.8 (3.5)
Sex				
Male	162 (48.4)	12.8 (8.1)	16.5 (9.1)	3.7 (3.7)
Female	173 (51.6)	13.4 (7.8)	17.6 (9.4)	3.8 (3.2)
Age				
10–11	220 (65.7)	13.0 (8.1)	17.0 (9.3)	3.9 (3.5)
12–13	115 (34.3)	13.3 (7.7)	17.2 (9.3)	3.5 (3.3)
Ethnicity				
Non Māori	113 (33.7)	12.6 (7.9)	16.1 (8.7)	3.8 (3.3)
Māori	222 (66.3)	13.4 (8.0)	17.5 (9.6)	3.8 (3.5)
NZDep.13				
High	257 (76.7)	13.1 (8.0)	17.2 (9.5)	3.7 (3.3)
Medium	62 (18.5)	13.3 (7.6)	16.9 (8.9)	4.2 (4.0)
Low	10 (3.0)	12.1 (7.1)	16.3 (6.8)	3.3 (2.0)
Caries experience				
Caries-free (DMFT/dmft = 0)	102 (30.4)	13.0 (7.8)	14.6 (8.6)	3.5 (3.7)
High (DMFT/dmft ≥ 4)	73 (21.8)	13.8 (7.8)	16.2 (8.0)	4.2 (3.7)

**Table 4:** Mean CPQ_11–14_, COHIP-SF and CARIES-QC scores by their global oral health questions (brackets contain standard deviations).

	**Self-Rated Oral Health**		
	Excellent/Very good	Good	Fair/Poor
Mean CPQ_11-14_	10.2 (7.7)	13.7 (7.5)	16.3 (7.8) ^a^
	Excellent/Good	Average	Fair/Poor
Mean COHIP-SF	15.1 (9.0)	19.2 (7.8)	21.1 (10.5) ^a^
	**Impact on Quality of Life**		
	Not at all	Very little	Some/A lot/Very much
Mean CPQ_11-14_	9.3 (6.4)	13.3 (6.9)	18.1 (8.4) ^a^
	Not at all	A bit	A lot
Mean CARIES-QC	2.5 (2.5)	4.3 (3.4)	5.9 (5.0) ^a^

^a^*P* < 0.05.

**Table 5:** Attrition analysis: comparison of the sociodemographic characteristics and oral health-related quality of life (OHRQoL) scale scores of children followed up and not followed up (brackets contain column percentages unless otherwise indicated).

Characteristics	Baseline	Followed Up	Not Followed Up
Total	335 (100.0)	240 (71.6)	95 (28.4)
Sex			
Male	162 (48.4)	116 (48.3)	46 (48.4)
Female	173 (51.6)	124 (51.7)	49 (51.6)
Age			
10 to 11	220 (65.7)	157 (65.4)	63 (66.3)
12 to 13	115 (34.3)	83 (34.6)	32 (33.7)
Ethnicity			
Non-Māori	113 (33.7)	91 (37.9)	22 (23.2)
Māori	222 (66.3)	149 (62.1)	73 (76.8) ^a^
NZDep13			
High	253 (77.8)	172 (74.8)	76 (83.5)
Medium	62 (19.1)	49 (21.3)	15 (16.5)
Low	1 (3.1)	9 (3.9)	0 (0.0)
Mean OHRQoL score (SD)			
CPQ_11–14_	13.1 (8.0)	12.2 (7.6)	15.4 (8.4) ^a^
COHIP-SF	17.1 (9.3)	14.8 (7.9)	17.1 (9.9)
CARIES-QC	3.8 (3.5)	2.7 (2.8)	4.6 (3.9)

^a^*P* < 0.05.

**Table 6:** Mean overall scores at baseline and follow-up, with effect sizes.

OHRQoL Measure	Baseline	Follow-Up	Change	Effect Size	Effect-Size DESCRIPTION
CPQ_11–14_	12.2 (7.6)	10.1 (7.1) ^a^	2.1 (7.8)	0.3	Moderate
COHIP-SF	14.7 (8.2)	14.9 (7.9)	−0.2 (8.6)	0.0	None
CARIES-QC (raw score)	3.5 (3.2)	2.7 (2.8) ^a^	0.8 (2.8)	0.3	Moderate
CARIES-QC (interval)	5.5 (3.2)	4.5 (3.1) ^a^	1.0 (2.9)	0.3	Moderate

^a^*P* < 0.0001.

**Table 7:** Change in mean CPQ_11–14_, COHIP-SF and CARIES-QC scores by change in global question.

Change	CPQ_11–14_ (N)	COHIP-SF (N)	CARIES-QC Raw (N)	CARIES-QC Interval (N)
Improved	4.7 ^a^ (64)	0.06 (36)	1.6 ^a^ (59)	1.7 ^a^
Stayed the same	1.8 ^a^ (138)	−0.06 (140)	0.8 ^a^ (132)	1.1 ^a^
Got worse	−0.8 (33)	−1.2 (47)	−0.3 (45)	0.2

^a^*P* < 0.05.

**Table 8:** Systematic evaluation of the three scales against the SAC criteria.

SAC Criterion	Measure		
	16-item CPQ_11–14_	COHIP-SF	CARIES-QC
Conceptual model	Yes	Yes	Yes
Reliability			
Internal consistency	Acceptable	Acceptable	Acceptable
Test-retest	Acceptable	Acceptable	Acceptable
Validity	Acceptable	Acceptable	Acceptable
Responsiveness	Acceptable	Not demonstrated	Acceptable
Interpretability	Acceptable	Acceptable	Acceptable
Burden	Acceptable	Acceptable	Depends ^a^
Alternative forms	Yes	Yes	No
Cross-cultural applicability	Demonstrated ^b^	Demonstrated	Emerging

^a^ The need to compute the interval score adds a layer of complexity to the generation of scale scores; ^b^ On the basis of evidence from earlier studies, as well as the current one.
